# Comparative metabolomics of paired maternal–cord samples identifies pathway-level divergence

**DOI:** 10.1007/s11306-026-02528-z

**Published:** 2026-09-23

**Authors:** Ashley Van Winckel, Mrinalini Gupta, Rachel Klein, Jagadeesh Puvvula, Joseph M. Braun, Kurt D. Pennell, Emily A. DeFranco, Shuk-Mei Ho, Yuet-Kin Leung, Ann M. Vuong, Zana Percy, Priyanka Bhashyam, Raymund Lee, Dean P. Jones, Vilinh Tran, Dasom V. Kim, Shouxiong Huang, Aimin Chen, Katherine E. Manz

**Affiliations:** 1 Department of Environmental Health Sciences, School of Public Health, University of Michigan, Ann Arbor, MI USA; 2 Department of Biostatistics, Epidemiology and Informatics, Perelman School of Medicine, University of Pennsylvania, Philadelphia, PA USA; 3 Department of Epidemiology, Brown University, Providence, RI USA; 4 School of Engineering, Brown University, Providence, RI USA; 5 Department of Obstetrics and Gynecology, College of Medicine, University of Kentucky, Lexington, KY USA; 6 Department of Pharmacology and Toxicology, College of Medicine, University of Arkansas for Medical Sciences, Little Rock, AR USA; 7 Department of Epidemiology and Biostatistics, School of Public Health, University of Nevada Las Vegas, Las Vegas, NV USA; 8 Department of Psychiatry and Human Behavior, Warren Alpert School of Medicine, Brown University, Providence, RI USA; 9 College of Arts & Sciences, University of Pennsylvania, Philadelphia, PA USA; 10 Division of Pulmonary, Allergy, Critical Care and Sleep Medicine, Emory University, Atlanta, GA USA; 11 Immunology Program, Memorial Sloan Kettering Cancer Center, New York, NY USA; 12 Pathogen-Host Interaction Program, Texas Biomedical Research Institute, San Antonio, TX USA

**Keywords:** Metabolomics, Maternal, Neonate, Pregnancy, Birth

## Abstract

**Introduction:**

During pregnancy, the maternal and fetal metabolomes are linked and can influence one another through factors such as maternal diet. However, there is a lack of understanding of baseline maternal and neonate metabolomic similarities and differences.

**Objectives:**

To investigate whether, for typical dyads, maternal metabolomic profiles can serve as a proxy for fetal metabolomic profiles, we conducted a cross-sectional study among a cohort of 65 pregnant persons and their neonates.

**Methods:**

We collected maternal and cord blood at the time of the delivery and utilized high-resolution mass spectrometry to perform untargeted metabolomics on maternal (*n* = 65) and neonate (*n* = 65) samples. We performed principal component analysis of the detected features to assess differences between mothers and neonates. We then performed a metabolome-wide association study to identify significant features and subsequently performed pathway enrichment analysis to identify associated metabolic pathways.

**Results:**

We observed distinct separation between features detected in mothers and neonates from both negative and positive ionization data. Our pathway analysis indicated that maternal and neonate metabolomes differ with respect to five major classes of metabolism, with 16 pathways implicated in metabolic differences between mothers and neonates. We observed pathway-specific metabolomic differences in mothers only, for fatty acid and lipid metabolism, with neonate metabolomic differences driven by non-pathway specific alterations in the global fetal metabolic profile.

**Conclusion:**

Our study provides evidence to support that maternal metabolomes are not proxies for fetal metabolomes and provides important insight on neonate metabolic changes in the window immediately before and after birth.

**Supplementary Information:**

The online version contains supplementary material available at https://doi.org/10.1007/s11306-026-02528-z.

## Introduction

Pregnancy involves the establishment of complex, interdependent, and dynamic physiological relations between the mother and the developing fetus. Changes in maternal diet, physical activity, and environmental exposures have been linked to fetal development and neonate outcomes and are known to occur through a combination of epigenetic, neurobiological, and physiological pathways (Monk et al., 2012). In turn, the developing fetus impacts the hormonal system, nutritional status, immune function, and brain structure of the mother (Dawe et al., 2007; Jee & Sawal, 2024; Monk et al., 2012; Waclawek & Park, 2023). This unique physiological state is reflected in metabolic changes for both mother and fetus. During pregnancy, maternal metabolism shifts to accommodate the needs of the growing fetus, while the fetus also develops its own metabolism. The maternal metabolome during pregnancy is significant, as it directly influences fetal development by providing essential nutrients and signaling molecules required for growth and development. For example, maternal triglyceride and glucose levels typically increase during pregnancy, enabling a ready supply of glucose for the fetus to support fetal growth and development (Wang et al., 2025). Conversely, the fetal metabolome encompasses the unique set of metabolites present in the developing fetus, reflecting its metabolic activities and health status but also deeply linked to the maternal metabolome. The physiological connection between a mother and her neonate is primarily facilitated through the placenta, a critical organ that allows for the passage of nutrients from mother to child through the umbilical cord. The maternal metabolome plays a pivotal role in shaping the fetal environment, as metabolites produced by the mother can cross the placental barrier and influence fetal growth and development, and essential nutrients, such as glucose, amino acids, and fatty acids, are transferred from the mother for fetal tissue development.

Over the past several decades, advances in bioinformatics tools and analytical chemistry technology have enabled analysis of biochemical pathways at the metabolite level. Metabolites encompass molecules such as lipids, amino acids, sugars, and nucleotides that have critical roles in biological processes, including energy production, cell signaling, and maintaining homeostasis, while the metabolome represents every small-molecule, or metabolite, in a biological sample (Schrimpe-Rutledge et al., 2016). While many studies have utilized metabolomics to identify specific metabolic biomarkers and link them to clinical outcomes, few studies to date have attempted to establish baseline metabolic profiles for mother-neonate dyads at birth to understand how maternal and neonate metabolomes differ and whether the maternal metabolome can be considered a true proxy for the fetal metabolome. While there is some evidence exploring baseline metabolic profiles and their similarities and differences in mother-neonate dyads (Perrone et al., 2020), there is a paucity of data which elucidates the specific pathways or metabolites for which the maternal and neonate metabolomes diverge, in large part due to the challenges of collecting neonate and maternal samples simultaneously during birth.

Metabolomics has become a powerful, discovery-based technique that can provide insights into disease states, reveal biomarkers for early diagnosis, and suggest potential targets for therapeutic interventions. For mother-neonate dyads, metabolomics has been used to link maternal health to fetal development and neonate health outcomes for a variety of clinical states and environmental exposures. This analytical technique has frequently been used in tandem with other statistical tools such as metabolome-wide association studies (MWAS) and pathway enrichment analysis to better understand and identify metabolic pathways associated with human exposure or disease. With advanced techniques such as untargeted metabolomics, coupled with powerful computational biology tools, researchers have also been able to identify new metabolites, as well as link unknown structures to critical metabolic pathways. For example, Zhai et al. (2023) used untargeted metabolomics to compare mother-infant dyads and identify potential metabolic pathways implicated in low infant birthweight. Other studies have linked maternal exposure to endocrine-disrupting chemicals to neonate endocrine function and well-being (Meeker, 2012), maternal nutrition and the child’s long-term disease risk (Marshall et al., 2022), and more. During pregnancy and the perinatal stage, both mother and child are particularly vulnerable; thus, understanding the differences and similarities in the metabolomic profiles of mothers and neonates is urgently needed. By developing a better understanding of how mother and neonate metabolomes may differ, we offer insight into this complex relationship, informing future research on phenotype etiologies, and establishing a much-needed foundation for research and development of clinical interventions in perinatal care for mothers and infants at this critical time point.

The objective of this study was to compare the maternal and fetal metabolomes at birth and to examine whether, for typical dyads, maternal metabolomic profiles can serve as a proxy for fetal metabolomic profiles. Additionally, we sought to explore how much variation observed in metabolomic differences is shared between mother-neonate dyads and which biological pathways are shared or unique at birth. Using untargeted metabolomics, we identified global metabolic differences between mothers and neonates, providing new insights into the distinct molecular mechanisms present at the time of delivery. Understanding these distinctions is crucial for advancing maternal and child health, as it can reveal how maternal metabolism directly influences fetal development and identify specific areas of the metabolome that are critical for healthy pregnancy outcomes.

## Method

### Study participants and sample collection

We recruited 75 pregnant people between August 2014 and September 2017 from the labor and delivery unit at the University of Cincinnati Medical Center, Cincinnati, Ohio. Participants were aged 18–45 years old and were recruited according to whether they were presenting for delivery at the time of the study. Participants were screened against additional exclusion criteria, of which a detailed description was previously published (Puvvula et al., 2023). Additional data relating to maternal and infant demographics, medical history, and pregnancy outcome was obtained from questionnaires and medical record reviews. During the hospital visit, each mother had a full-term, live singleton delivery. From each mother-infant dyad, 15 mL of maternal venous blood and 10mL of venous cord blood was collected at the time of delivery.

IRB approval for the study was obtained from the University of Cincinnati Institutional Review Board (IRB 2013–4772) (Puvvula et al., 2023). Written informed consent was obtained from each study participant at the time of study enrollment.

### High-resolution metabolomics

We acquired high-resolution metabolomics data using previously established liquid chromatography high-resolution mass spectrometry (LC-HRMS) methods. Briefly, we isolated 65-uL serum aliquots from maternal and cord blood samples and stored the serum at −80 °C. We then performed sample extraction of the frozen serum aliquots by thawing them on ice and adding 130 µL of acetonitrile containing a mixture of stable isotope standards, followed by sample vortexing, equilibrating for 30 min, and centrifuging. We transferred the resulting monophasic supernatant to an LC-HRMS analysis vial and analyzed the extracts on a Q-Exactive HF orbitrap mass spectrometer equipped with a Dionex Ultimate 3000 chromatograph (Thermo Scientific). For each sample, we analyzed 10 µL aliquots in triplicate using both forward and reverse phase chromatography in positive and negative ionization modes to increase metabolite feature coverage. For forward phase chromatography, we employed a hydrophilic interaction liquid chromatograph (HILIC) column with the electrospray ionization (ESI) source operated in positive mode. For the reverse phase chromatography, we employed a C-18 column with the ESI source operated in negative mode. Mobile phases for analyte separation consisted of formic acid, acetonitrile, and UHPLC-MS grade water. We operated the high-resolution mass spectrometer in full scan mode at 120,000 resolution and a mass-to-charge ratio (*m/z)* range of 85 to 1,275. Additional information regarding chromatography and scan parameters are provided in the Supporting Information. To preclude batch effects, we randomized and split the maternal and neonate samples into four batches of 24 to 47 samples. Maternal and neonate samples were not paired in analytical batches. Additionally, we analyzed quality control (QC) samples, including standard reference materials (SRMs; NIST 1950) and a pooled plasma sample at the beginning, middle, and end of each batch, and isotopically labeled internal standards were used to evaluate injection consistency and method performance in real time.

### Data preprocessing

We saved spectral files in the.RAW file format and converted them to.cdf format using XCalibur File Converter. We then extracted and aligned the converted files using apLCMS to obtain uniquely detected ions, or features, characterized by their respective *m/z*, retention time, and ion abundance. We were able to achieve detection of 10,207 features in the positive ionization mode and 6,857 features in the negative ionization mode. We evaluated triplicate injections using xMSanalyzer to determine the coefficient of variation (CV). We excluded features with a CV ≥ 30% from the feature table and summarized the remaining triplicate injections according to their median value. We additionally excluded features with ≥ 20% missingness (i.e., ion was not detected) to improve the reliability of downstream statistical analyses. To preclude batch effects, we also performed batch correction using the ComBat algorithm in xMSanalyzer.

### Statistical analysis

We performed all statistical analyses using RStudio Version 4.3.3 (R Core Team, 2023). We computed the descriptive statistics for the sample, including median, interquartile range (IQR), and count (frequency) where appropriate. We excluded unpaired samples (i.e., maternal samples without a neonate pair) from analysis and one participant for whom data was missing (*n* = 10).

Prior to principal component analysis (PCA), we log_2_-transformed feature area counts to approximate normality assumptions for subsequent regression models. We performed dimensionality reduction and feature count comparison through PCA using the R function *prcomp* to generate biplots of the similarities and differences in maternal and neonate metabolomes. Prior to PCA plot generation, we imputed any missing values as the minimum value for the row (i.e., for the feature) divided by the square root of two, as PCA cannot handle missing data. We performed PCA for mother-neonate dyads separately for positive and negative ionization modes. The R package *ggplot2* was used to visualize the results of the PCA. To assess clustering, the function *stat_ellipse* was used to draw 95% confidence ellipses (assuming a multivariate *t*-distribution) for each mode, respectively. We also generated loadings plots for negative and positive ionization features using the R package *factoextra* for the top 20 features contributing to PC1 and PC2.

### Metabolome-wide association study (MWAS)

To evaluate whether the difference in intensity of the detected features was correlated with status (i.e., mother or neonate), we performed an MWAS. We constructed a linear mixed-effects model using paired maternal-neonate samples. We used a random intercept for the identification number of the mother-neonate pair and a fixed slope for the sample strategy (i.e., whether the sample group was maternal or neonate), with feature intensity serving as the outcome. We adjusted the linear model for five covariates that were identified a priori as potential confounders and obtained from a combination of questionnaires and medical record reviews—mother’s age (continuous), smoking status (yes or no), delivery type C-section (yes or no), neonate sex (female or male), and number of prior pregnancies (discrete). Linear models were applied separately for features detected in positive and negative ionization modes. The outcomes from each regression model were combined into one dataset for FDR calculation. The false discovery rate (FDR) was controlled using the Benjamini-Hochberg method at a threshold of 20% (Benjamini & Hochberg, 1995). This threshold was selected due to the exploratory nature of our analysis and to preserve sensitivity in the context of a modest sample size and highly correlated metabolomic features while controlling the expected proportion of false discoveries among identified features. We created volcano plots to visualize the feature count differences between the mother-neonate pairs. The effect direction was used to identify whether significant features (q < 0.20) were higher in mothers or neonates.

### Pathway analysis

To determine which pathways were significantly different between mothers compared to neonates, we performed pathway analysis of the identified features. The combined dataset of positive and negative ionization features significant at the FDR-adjusted p-value (q < 0.20) were uploaded with their p-values, t-scores, and retention times to MetaboAnalyst R 5.0. We applied the *mummichog* algorithm (version 2.0) with a cutoff for the top 10% features based on the ranking of p-values from the MWAS using 10,000 permutations. Features were compared against the *Homo sapiens* MFN pathway library, with only pathways containing at least three metabolites considered for analysis. Final metabolic pathways were considered statistically significant if the Gamma was below 0.05. Pathways identified as enriched in the final set were grouped by similarity of metabolic pathways according to the KEGG BRITE database. Additionally, secondary exploratory pathway analyses were performed on subsets of the dataset. The features higher in mothers and higher in neonates were separated into two subsets, and the *mummichog* algorithm was applied to both subsets individually in the same manner described above for the combined dataset.

## Results

### Sample characteristics

Of the 75 original pregnant persons recruited, we included 65 mother-neonate pairs in the MWAS, after excluding participants without a neonate pair and one participant with missing sample data (*n* = 10). The mothers had a median age of 28.5 years (IQR: 25.0 − 32.0) at the time of delivery, who primarily identified as African American (47.7%) or non-Hispanic White (40.0%), and 20% reported having smoked at least once during their pregnancy (*n* = 13). The majority (66.1%) reported three or fewer previous pregnancies, and the median number of pregnancies was 2 (IQR 0.5 − 3.5). The majority of mothers (63.1%) reported delivery via cesarean-section. Of the neonates, 49.2% were female (*n* = 32). Additional sample characteristics are presented in Table 1 in Supporting Information.

### Feature detection

In total, we detected 17,064 features across all 130 samples; 6,857 of which were detected in negative ionization mode and 10,207 in positive ionization mode. After applying log2 transformation to the data and filtering for features detected in at least 80% of the samples, we included 3,641 and 5,244 features in further statistical analysis for negative and positive ionization, respectively.

### Principal component analysis (PCA) 

**Fig. 1:**
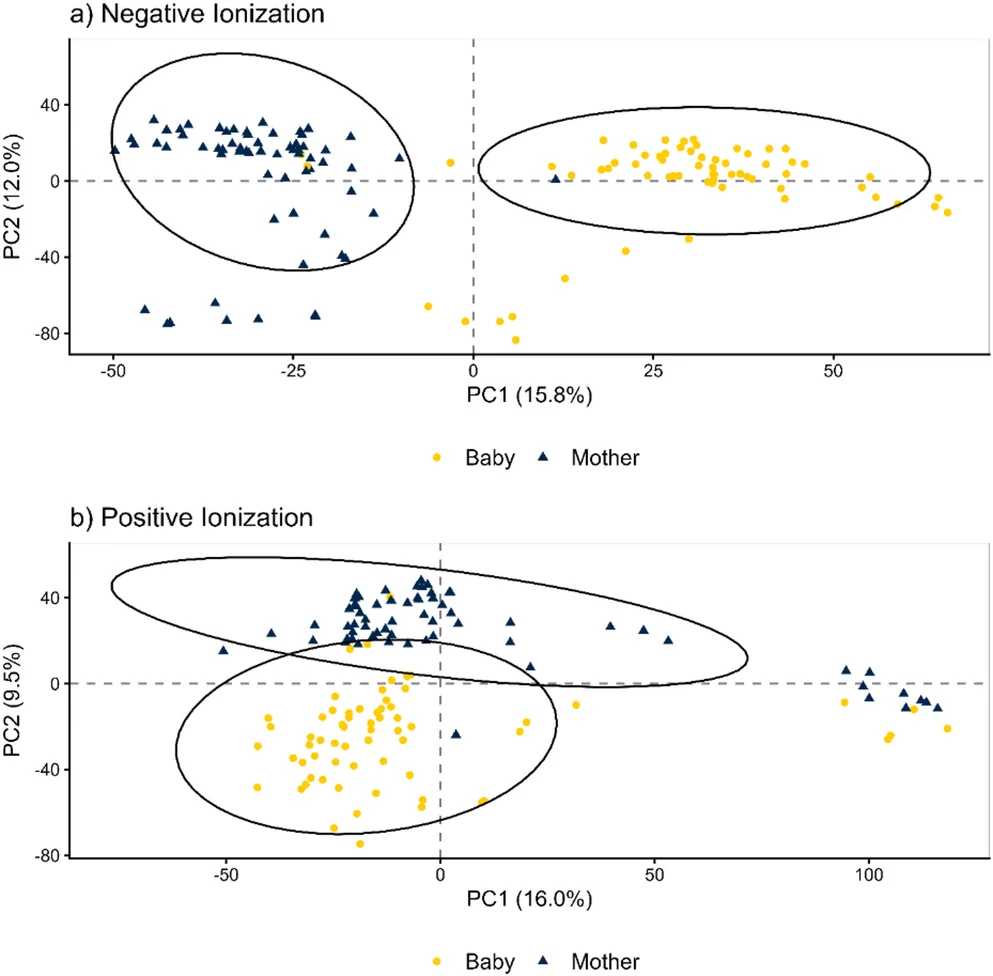
**a** Principal component analysis of features detected in negative ionization mode for mothers and neonates. **b** Principal component analysis of features detected in positive ionization mode for mothers and neonates

We performed PCA to evaluate the variability between the two sample groups (i.e., mothers and neonates) respectively, for negative and positive mode feature data. For features detected in negative ionization mode, we observed distinct clustering and separation of the mother group and the neonate group (Fig. 1(a)). For negative features, PC1 explained 15.8% of the variation in the detected features, while PC2 explained 12.0% of the variation in the detected features.

**Fig. 2:**
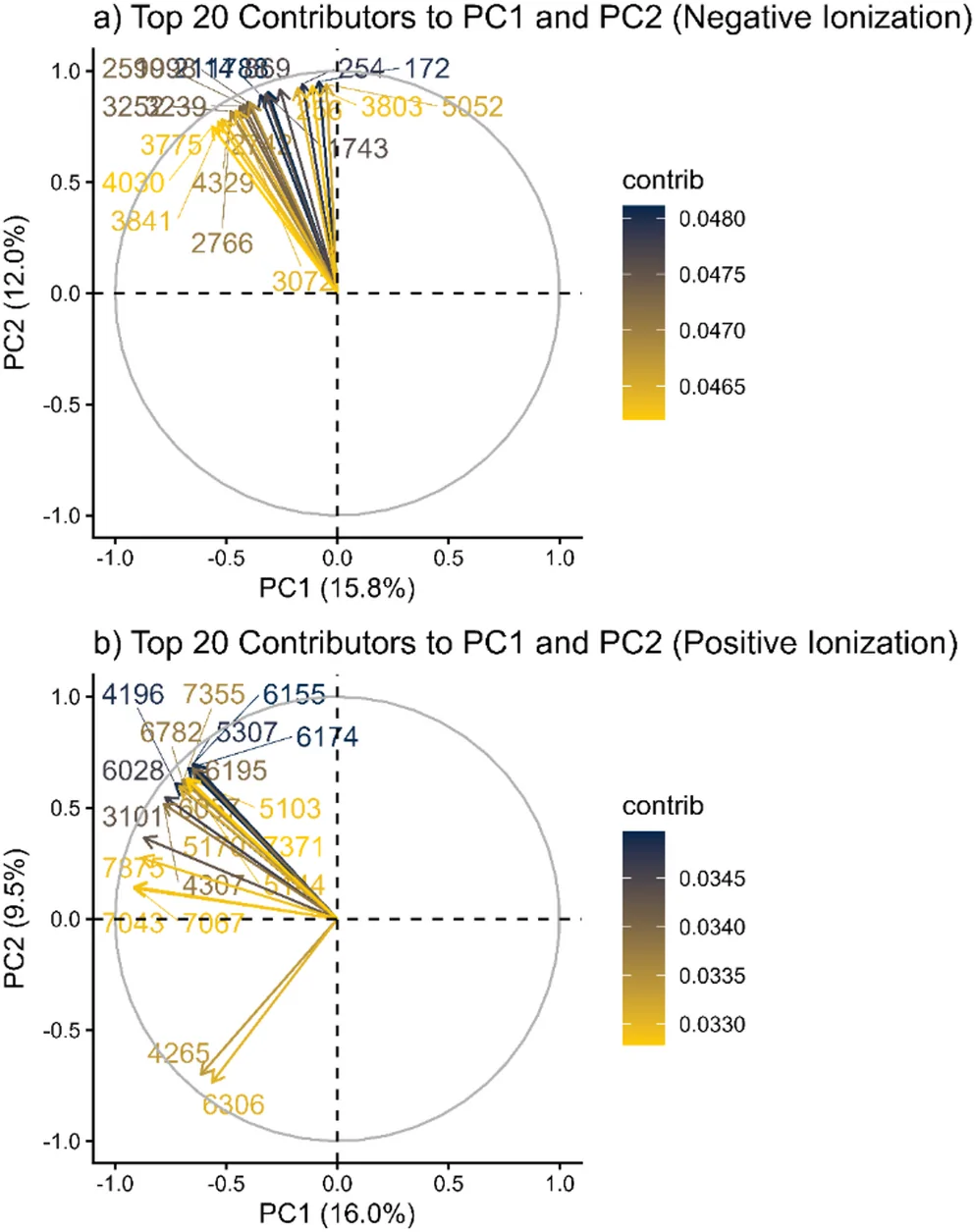
a. Loadings plot of the top 20 features contributing to PC1 and PC2 for features detected in negative ionization mode. **b** Loadings plot of the top 20 features contributing to PC1 and PC2 for features detected in positive ionization mode

We also observed clustering according to the sample group (i.e., mother or neonate) for features detected in positive ionization mode (Fig. 1(b)). This was less marked than for negative features, with some overlap between the two groups. PCs 1 and 2 explained 16.0% and 9.5% of the variance between the sample groups, respectively. Loadings plot for the top 20 features contributing to PC1 and PC2 for both modes (Fig. 2) are also shown. The high dimensionality of the data, an inherent quality of untargeted metabolomics data, may have contributed to greater overlap of the sample groups observed on the PCA plot. Additionally, we observed a group of outliers (*n* = 14) in positive mode; the sample and analysis characteristics for these participants are presented in Fig. 7 of Supporting Information. As there was no indication that outlier status was due to sample analysis error or batch bias, all samples were included in downstream statistical analyses. Overall, our PCA findings indicate that mothers and neonates exhibit distinct separation with respect to metabolic features detected in both negative and positive ionization modes.

### Metabolome-wide association study (MWAS)

**Fig. 3:**
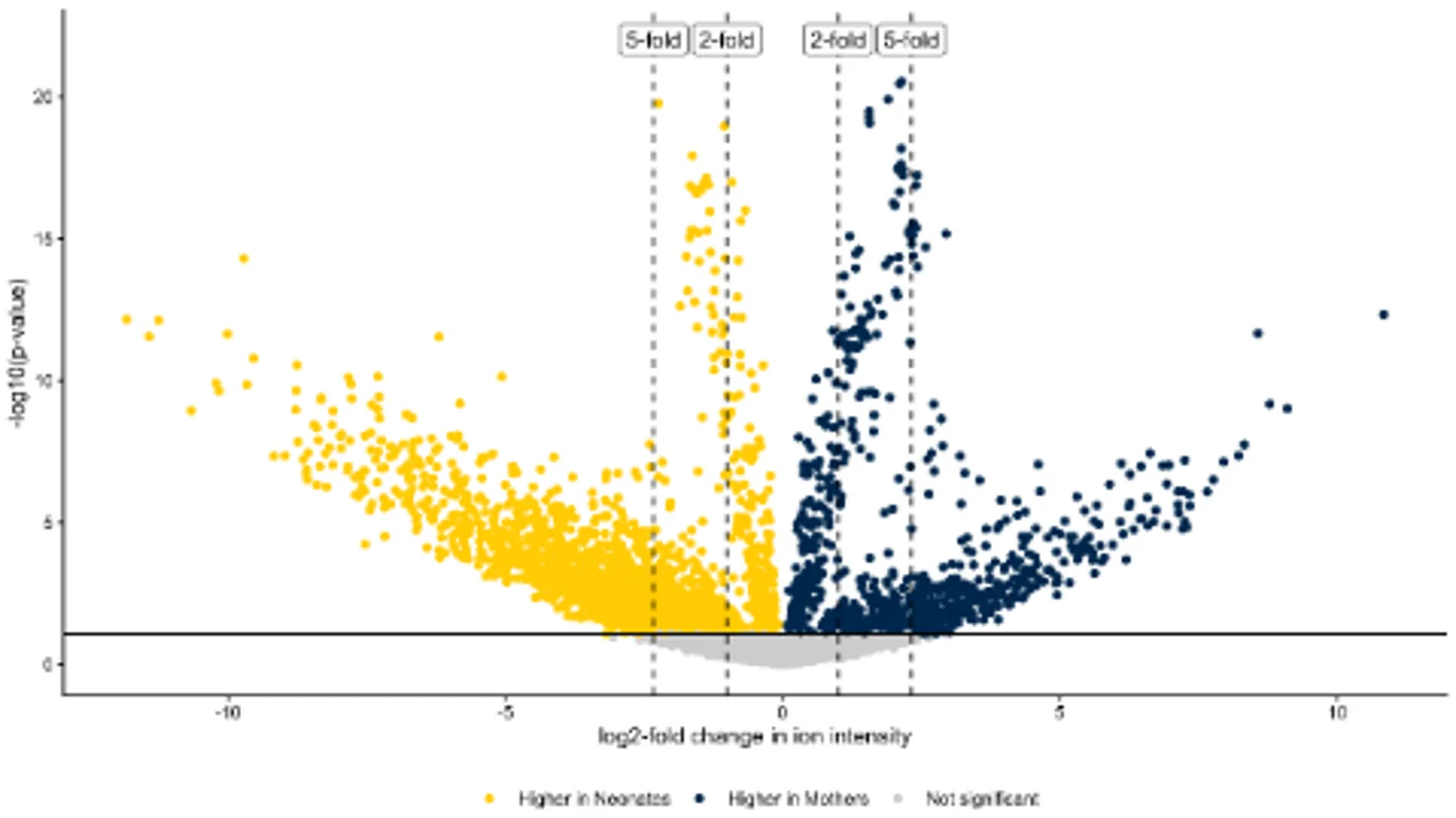
Metabolome-wide association study significant features (positive and negative ionization mode) for mothers and neonates

We performed an MWAS to determine which features differed significantly between mothers and neonates (Fig. 3). Mothers were compared against neonates, with “negative” estimates reflecting higher intensity of that feature in neonates and “positive” estimates reflecting higher intensity of that feature in mothers. Of the features detected, a total of 3,720 significant features were detected (41.9%). For features detected in negative ionization mode, 1,766 features were significant (48.5%, q < 0.20). Of the significant features, 1,086 were higher in babies (61.5%), and 680 were higher in mothers (38.5%). For features detected with positive ionization mode, 1,954 features were significant (37.3%, q < 0.20). Of the significant features, 1,669 were higher in babies (85.4%) and 285 were higher in mothers (14.6%).

### Pathway analysis findings

#### All significant features

**Fig. 4:**
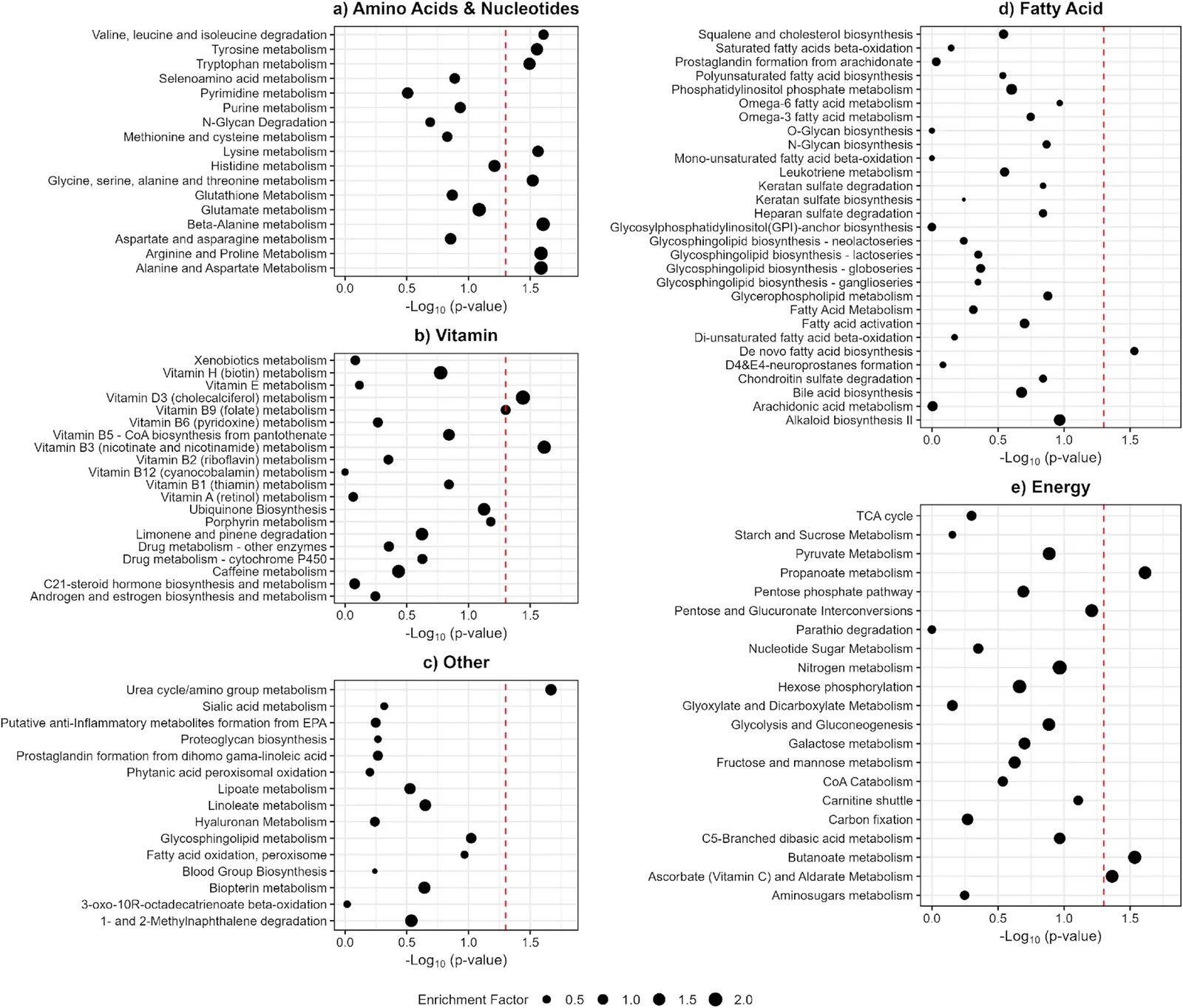
Pathway analysis results for positive and negative features (*n* > 3) for all significant features detected in the MWAS. Values to the right of the vertical dashed red line are significant at *p* < 0.05

The first pathway analysis included all significant features detected in the MWAS, both higher in mothers and higher in neonates, allowing for broad characterization of the pathways implicated in metabolic differences in mother-neonate dyads, in comparison to the null distribution. The results of our pathway analysis (Fig. 4) found that the observed differences in metabolome-associated feature intensities mapped onto 16 unique pathways. We further grouped these metabolic pathways into five classes: amino acids and nucleotides, vitamins, lipids and fatty acids, energy, and other, with at least one pathway implicated in each major metabolic class. For the amino acids and nucleotides, of the 17 metabolic pathways determined as significant in the MWAS, eight pathways were significantly different than the null: valine, leucine, and isoleucine degradation, tyrosine metabolism, tryptophan metabolism, lysine metabolism, glycine, serine, alanine, and threonine metabolism, beta-alanine metabolism, arginine and proline metabolism, and alanine and aspartate metabolism. In the energy group, of the 21 pathways that were significant in the MWAS, three pathways were significantly different: propanoate metabolism, butanoate metabolism, and ascorbate and aldarate metabolism. For the vitamins group, of the 20 pathways that were significant in the MWAS, three pathways were significantly different: vitamin D3 metabolism, vitamin B9 metabolism, and vitamin B3 metabolism. In the lipids and fatty acids group, of the 29 pathways that were significant in the MWAS, one pathway was significantly different: de novo fatty acid synthesis. Finally, in the other group, of the 15 pathways identified as significant in the MWAS, one pathway was significantly different: urea cycle/amino group metabolism.

#### Pathway analysis of significant features higher in mothers

**Fig. 5:**
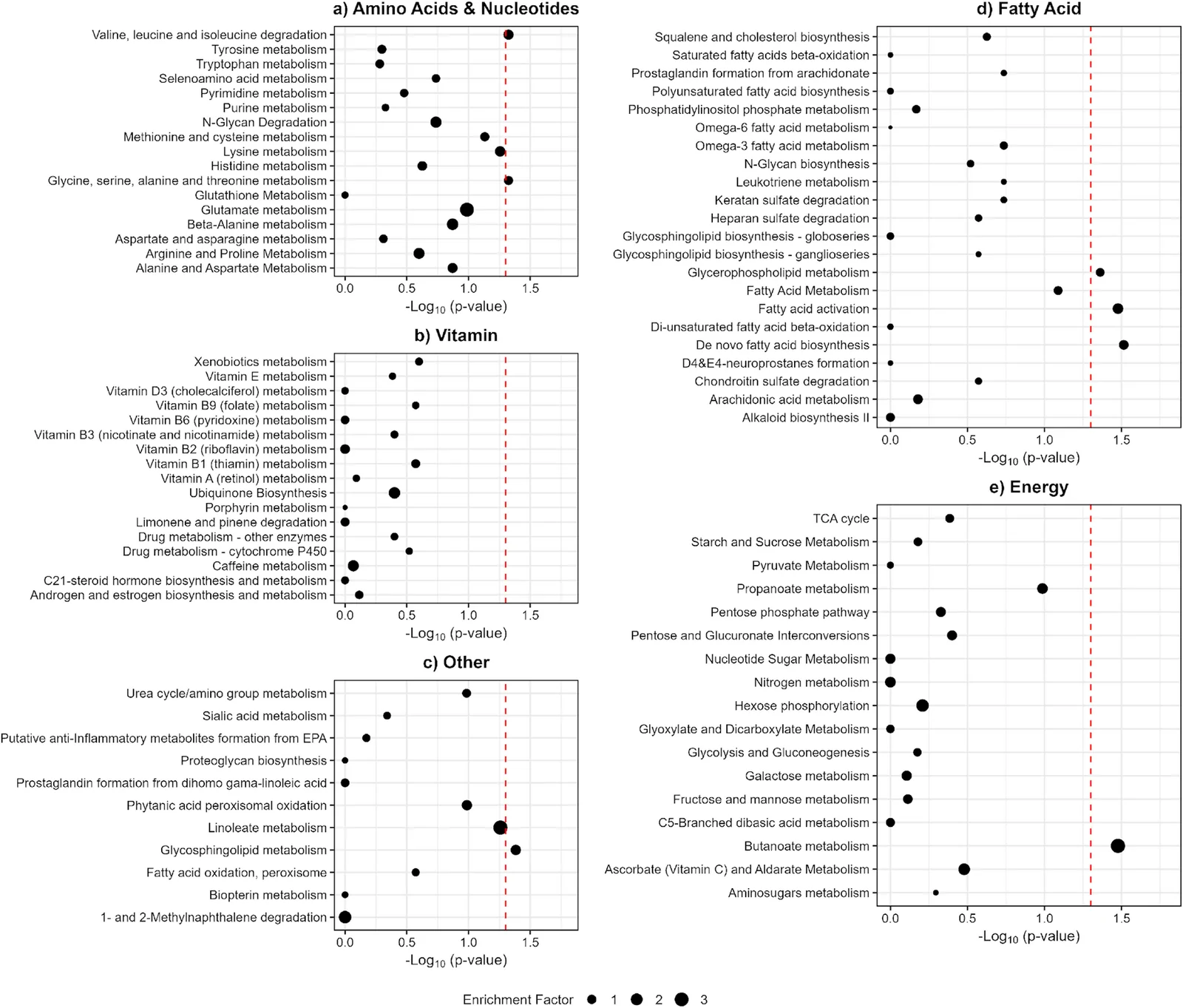
Pathway analysis results for positive and negative features (*n* > 3) for features significantly higher in mothers compared to neonates. Values to the right of the vertical dashed red line are significant at *p* < 0.05

We performed additional exploratory analyses to isolate the role of differential pathway enrichment between mothers and neonates. After sub-setting and analyzing the features that were higher in mothers only, we found that seven pathways were significantly enriched in mothers (Fig. 5). For the amino acids and nucleotides, of the 17 metabolic pathways determined as significant in the MWAS, two pathways were significantly enriched: valine, leucine, and isoleucine degradation, and glycine, serine, alanine, and threonine metabolism. In the energy group, of the 21 pathways that were significant in the MWAS, one pathway was significantly enriched, butanoate metabolism. For the vitamins group, of the 20 pathways that were significant in the MWAS, none were significantly enriched. In the lipids and fatty acids group, of the 29 pathways that were significant in the MWAS, three pathways were significantly enriched: glycerophospholipid metabolism, fatty acid activation, and de novo fatty acid biosynthesis. Finally, in the other group, of the 15 pathways identified as significant in the MWAS, one pathway was significantly enriched: glycosphingolipid metabolism.

#### Pathway analysis of significant features higher in neonates

**Fig. 6:**
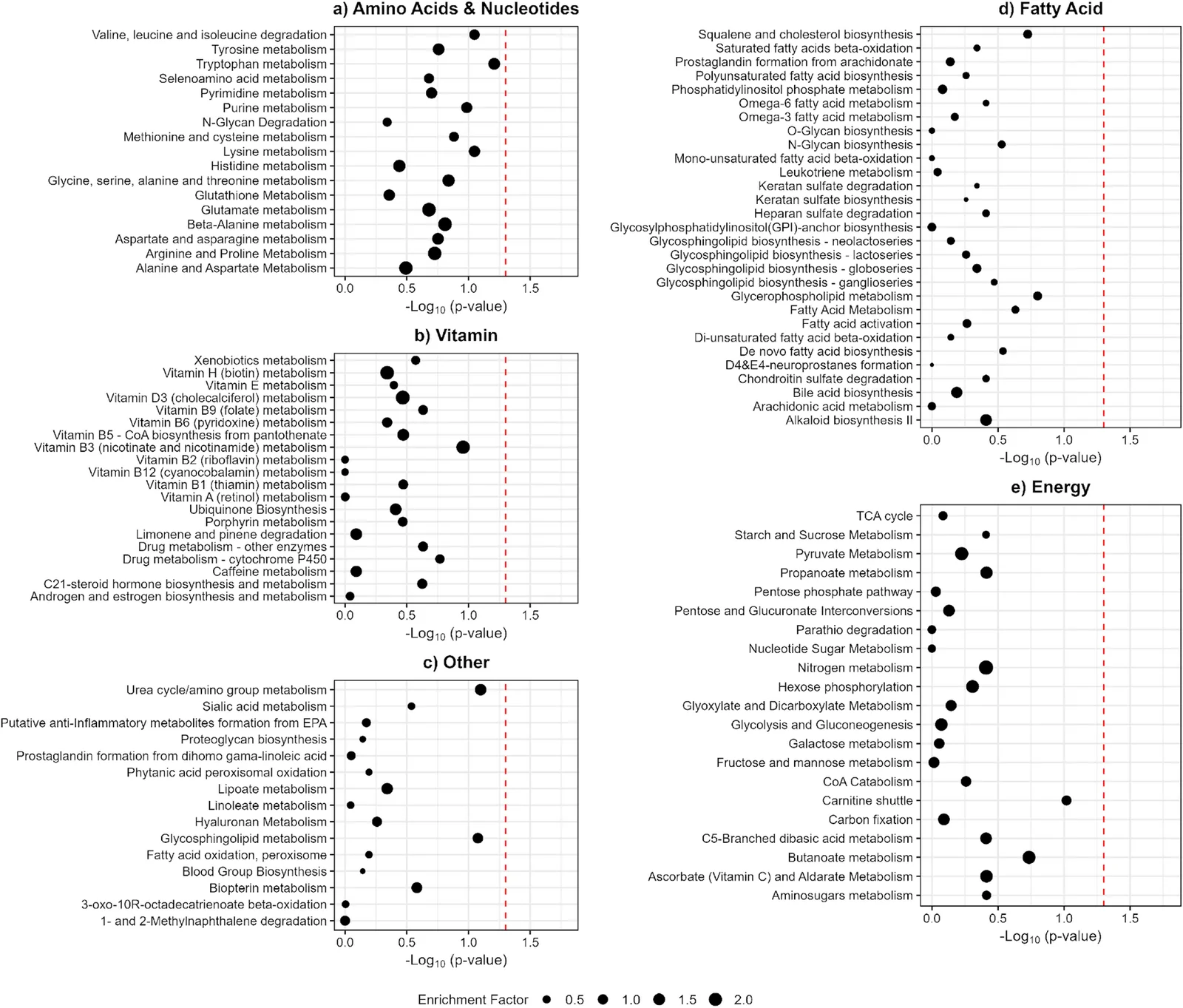
Pathway analysis results for positive and negative features (*n* > 3) for features significantly higher in neonates compared to mothers. Values to the right of the vertical dashed red line are significant at *p* < 0.05

The same pathway analysis was applied to the subset of features identified in the MWAS that were significantly higher in neonates compared to mothers (Fig. 6). Of the five metabolic classes and 102 metabolic pathways represented by those classes, no significantly enriched pathways were observed.

## Discussion

In this study, we characterized the differences between mother and neonate metabolic profiles by utilizing an untargeted mass spectrometry approach. The results of our PCA showed distinct differences for mother-neonate dyads at birth, with our MWAS and pathway analysis indicating which metabolites and metabolic pathways were significantly different in these mother-neonate dyads. Significant metabolites higher in either mothers or neonates were associated with 16 different significantly enriched pathways across five major classes of metabolic functions. When significant metabolic features higher in mothers alone were mapped, seven metabolic pathways were significantly enriched; conversely, no pathways were significantly enriched for features higher in neonates alone. Current knowledge suggests that the maternal metabolome has a substantial, if not partially deterministic role, in fetal metabolomic changes and subsequent outcome and that divergence of mother and fetal metabolome profiles occurs after birth, with little understanding of how these profiles differ or are similar at birth. By collecting cord blood within minutes of delivery, our findings provide insight into the metabolic profiles of mothers and neonates prior to these transitional stages, highlighting for which metabolites and metabolic pathways neonate and maternal metabolomes differ significantly at the time of birth and potentially capturing metabolic changes immediately before or during birth. Additionally, the observed differences in maternal and neonatal metabolomic profiles suggest that the maternal metabolome may not fully capture the fetal or newborn metabolic profile, limiting diagnostic and clinical use for maternal metabolomes as a proxy for fetal metabolomes.

Our findings are consistent with existing knowledge about maternal metabolism during pregnancy. Throughout fetal development, the primary energy source of the fetus is maternal and placental glucose. The mother undergoes major metabolic changes to accommodate this need, such as increased glucose production and insulin resistance, in order to supply the additional energy required for the growth and maintenance of the fetus, placenta, and maternal tissue (Berggren et al., 2016; Stuebe & Rich-Edwards, 2009; Tinius et al., 2021). This phenomenon may explain our observation of significant alterations in metabolites for pathways related to energy metabolism, especially for significantly enriched pathways in maternal metabolic profiles. The significantly enriched metabolic pathways we observed in this study relate to known or previously observed pregnancy-induced physiological changes, including fatty acid synthesis (Carreras-Badosa, 2016), glycosphingolipid metabolism (Lantzanaki et al., 2023), and other metabolic changes associated with fatty acids and lipid metabolism. After birth, the neonate must make a quick transition from the continuous supply of glucose from the mother to a new supply of energy (Ward Platt & Deshpande, 2005). Previous studies have observed metabolic changes and significant neonate metabolic development in the first several hours to days of life (Georgakopoulou et al., 2020). Our findings of no significantly enriched metabolic pathways associated with features higher in neonates is consistent with observed timelines of neonatal development.

Maternal and fetal metabolomes also differ with respect to response to and metabolism of toxicants. Further studies from this cohort suggest that maternal and fetal metabolomes diverge with respect to metabolic changes in response to chemical exposures, including polycyclic aromatic hydrocarbons (PAH) (Puvvula et al., 2023). and endocrine-disrupting compounds (EDC) (Puvvula et al., 2025). Our group has also compared maternal and fetal metabolome data (in the same cohort as the present study) in relation to maternal urinary PAH concentrations. We found that most metabolic pathways associated with PAH exposure (43 out of 59) were distinct between mothers and their neonates, with little overlap between neonates and mothers. In mothers, PAH concentration-dependent changes were primarily associated with lipid, fatty acid, and xenobiotic and carbohydrate metabolic pathways. In contrast, PAH concentration-dependent changes in neonate metabolites were primarily associated with lipid and vitamin metabolic pathways. Additionally, PAH-related metabolic changes were induced differentially for different PAHs for mothers and neonates. In a separate study, our group also investigated changes in global serum metabolome profiles in maternal and neonate samples associated with gestational exposure to EDCs (paraben/phenol, phthalate, and phthalate replacements). While pathways associated with some EDCs showed overlap (e.g., BPS was primarily implicated in amino acid metabolism for both maternal and neonate metabolomes), different EDCs were associated with metabolite concentration in mothers compared to neonates. For example, pentose/glucuronate interconversions were enriched for neonates in association with maternal EDC concentrations, while lipoate metabolism, among others, was enriched at a higher magnitude in mothers. However, more overlap was observed for maternal and fetal metabolic changes in response to EDC exposure compared to PAH exposure. The findings of these three studies in tandem suggest that the maternal and fetal metabolomes diverge in respect to both typical metabolic function as well as in response to various gestational exposures.

### Strengths and limitations

Our study’s findings are supported by the collection of high-resolution HPLC-MS data using validated sample extraction techniques in two ionization modes, increasing data quality and coverage. Additionally, by performing the MWAS using a metabolomics model generated from multiple databases (*Homo sapiens* MFN) we reduced the potential for database bias in our MWAS results. However, several key limitations of our study exist, including the small sample size of the study cohort, unmeasured confounders, and the timing of the sample collection. The small sample size of our study cohort limits the power of our study and combined with the high dimensionality inherent in metabolomics datasets, may lead to the presence of bias that could inflate the occurrence of false positives; these concerns may be higher for the exploratory pathways analyses that attempt to elucidate intra-dyad metabolic differences. Furthermore, without knowledge of confounders such as the mother’s diet and unknown environmental exposures and their association with the observed mother-neonate differences, we cannot explain distinct variations in the paired metabolomes. Finally, due to the challenges in obtaining in-utero fetal blood samples, we are unable to determine at which point during pregnancy mother-fetus metabolomic divergence occurs. These limitations suggest the need for further research investigating these metabolomic differences in mother-neonate dyads.

## Conclusion

Our cross-sectional study identified the pathways which are significantly different between mothers and neonates at the time of birth, highlighting the differences in the metabolic profiles of these dyads. Our findings provide evidence to support that maternal metabolomes may not serve as adequate proxies for fetal metabolomes. Further studies are needed to identify mechanisms of these metabolic differences.

## Supplementary Information

Below is the link to the electronic supplementary material.

Supplementary Material 1

## Data Availability

The datasets generated and/or analyzed during the current study are not publicly available due to our extended ongoing analysis.
