# Supplementary material for: Comparative metabolomics of paired maternal–cord samples identifies pathway-level divergence
Source: Metabolomics. 2026 Sep 23;22(5):157. doi: 10.1007/s11306-026-02528-z (PMC13601144; doi:10.1007/s11306-026-02528-z)
Supplement: Supplementary file 1 [file 11306_2026_2528_MOESM1_ESM.pdf]

Supporting Information for
Comparative metabolomics of paired maternal–cord samples identifies pathway-level divergence

Ashley Van Winckel, Mrinalini Gupta, Rachel Klein, Jagadeesh Puvvula, Joseph M. Braun, Kurt D. Pennell, Emily A. DeFranco, Shuk-Mei Ho, Yuet-Kin Leung, Ann M. Vuong, Zana Percy, Priyanka Bhashyam, Raymund Lee, Dean P. Jones, Vilinh Tran, Dasom V. Kim, Shouxiong Huang, Aimin Chen, Katherine E. Manz

Corresponding author: Katherine E. Manz – Department of Environmental Health Sciences, School of Public Health, University of Michigan, Ann Arbor; email: katmanz@umich.edu

This file contains:

Fig 7 (page 3)
Tables 1 to 5 (pages 3-6)

Figures

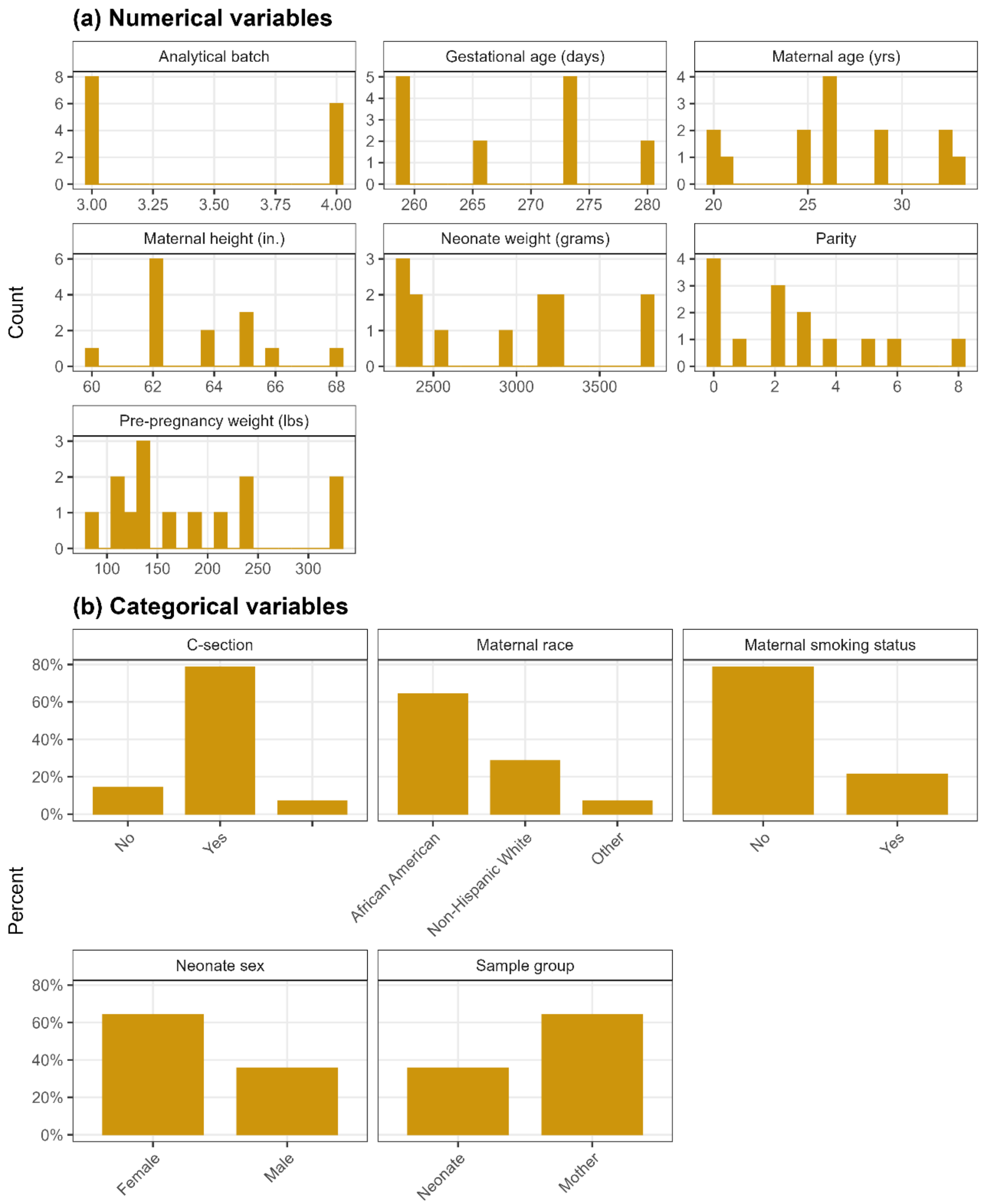

Fig 7. Characteristics for the participant sample outliers cluster identified in PCA of positive ionization mode features.

Tables

Table 1. Study population characteristics for the 65 participating pairs.

Variable	Value
Maternal age (years)[a]	28.5 (25.0-32.0)
Gestational age (weeks)[a]	39.0 (39.0-39.0)
Maternal pre-pregnancy weight[a]	72.6 (58.0-86.9)
Parity[b]	1: 11 (16.9)
	2: 15 (23.1)
	3: 9 (13.8)
	≥ 4: 16 (24.6)
Smoking during pregnancy[b]	Yes: 13 (20.0)
	No: 48 (73.8)
Race[b]	Non-Hispanic White: 26 (40.0)
	Non-Hispanic Black: 31 (47.7)
	Asian: 2 (3.1)
	Hispanic: 2 (3.1)
	Other: 4 (6.2)
Delivery via cesarean-section[b]	Yes: 41 (63.1)
	No: 19 (29.2)
Newborn sex[b]	Male: 33 (50.8)
	Female: 32 (49.2)

a-median and interquartile range & b-count and percentage

Table 2. R packages used in statistical analysis.

R package
ggplot2
gridExtra
plyr
stringr
dplyr
gtools
ggpubr
plotly
statmod
lmerTest
MetaboAnalystR
tibble
cowplot
factoextra

Table 3. Loadings scores across PC1 and PC2 for features detected in negative ionization mode.

Feature ID	m/z	Time	PC1	PC2	Combined contribution to PC1 & PC2
1788	240.9287	45.8	-0.00947	0.03156	0.04811
2114	263.9794	60.8	-0.01051	0.03110	0.04803
172	102.9375	44.5	-0.00252	0.03322	0.04802
254	112.9781	57.5	-0.00475	0.03286	0.04792
869	170.9443	45.3	-0.00784	0.03197	0.04764
1743	238.9318	42.9	-0.00977	0.03125	0.04760
1998	254.9443	43.3	-0.01229	0.03001	0.04748
3239	344.9793	57.8	-0.01335	0.02938	0.04738
3252	345.9822	57.1	-0.01461	0.02856	0.04737
2590	296.8903	42.6	-0.01287	0.02962	0.04730
2766	308.9160	44.0	-0.01461	0.02844	0.04705
4329	426.9824	58.1	-0.01465	0.02835	0.04690
2742	306.9192	43.7	-0.01196	0.02991	0.04677
5052	496.9281	63.6	-0.00150	0.03278	0.04654
256	112.9931	52.9	-0.00544	0.03219	0.04643
3072	330.9635	61.1	-0.01389	0.02866	0.04641
3841	388.9223	47.0	-0.01577	0.02731	0.04635
3775	384.9720	51.6	-0.01629	0.02688	0.04628
3803	386.9295	53.2	-0.00344	0.03250	0.04628
4030	402.9380	44.3	-0.01712	0.02615	0.04620

Table 4. Loadings scores across PC1 and PC2 for features detected in positive ionization mode.

Feature ID	m/z	Time	PC1	PC2	Combined contribution to PC1 & PC2
6155	400.8269	82.3	-0.01607	0.02247	0.03498
6174	402.8238	82.5	-0.01658	0.02181	0.03495
4196	278.8751	82.5	-0.01807	0.01965	0.03487
5307	344.8654	82.8	-0.01613	0.02229	0.03482
6028	390.7944	79.8	-0.01917	0.01759	0.03460
3101	217.9260	86.3	-0.02154	0.01173	0.03427
6195	404.8209	82.7	-0.01613	0.02178	0.03397
4307	284.9098	89.4	-0.01935	0.01664	0.03381
6782	462.7783	81.4	-0.01728	0.02010	0.03378
7355	518.7395	80.2	-0.01711	0.02021	0.03359
6057	392.7951	82.2	-0.01719	0.02008	0.03357
4265	283.0059	82.3	-0.01519	-0.02254	0.03337
5170	336.8337	82.3	-0.01759	0.01921	0.03315
6306	414.8963	82.9	-0.01395	-0.02368	0.03306
5144	334.8366	82	-0.01680	0.02024	0.03295
7875	584.6401	79.1	-0.02186	0.00883	0.03292
5103	332.8396	81.3	-0.01671	0.02033	0.03290
7043	488.8721	94.6	-0.02263	0.00443	0.03290
7067	490.8691	93.7	-0.02259	0.00465	0.03286
7371	520.7373	81.5	-0.01674	0.02021	0.03278

Table 5. Pathway analysis parameters.

**Parameter**	
Ranking criterion	P-value
Mass tolerance	5 ppm
Ion mode	Both
Enforce primary ions	No
Edit adducts	Yes, custom
Algorithm	HSA_MFN
MetaboAnalyst Version	6.0
P-value cutoff	p(Gamma) < 0.05
Pathway library	KEGG, BiGG, Edinburgh
Number of metabolites in each pathway	≥ 3
Permutations	10,000
Adducts	M+FA-H [1-], M-H [1-], 2M-H [1-],M-H+O [1-], M(C13)-H [1-], 2M+FA-H [1-], M-3H [3-],M-2H [2-], M+ACN-H [1-], M+HCOO [1-], M+CH3COO [1-], M-H2O-H [1-], M [1+], M+H [1+], M+2H [2+], M+3H [3+], M+H2O+H [1+], M-H2O+H [1+], M(C13)+H [1+], M(C13)+2H [2+], M(C13)+3H [3+], M-NH3+H [1+], M+ACN+H [1+], M+ACN+2H [2+], M+2ACN+2H [2+], M+3ACN+2H [2+], M+NH4 [1+], M+H+NH4 [2+], 2M+H [1+], 2M+ACN+H [1+])
